# A piezoresistive-based 3-axial MEMS tactile sensor and integrated surgical forceps for gastrointestinal endoscopic minimally invasive surgery

**DOI:** 10.1038/s41378-024-00774-6

**Published:** 2024-09-27

**Authors:** Cheng Hou, Huxin Gao, Xiaoxiao Yang, Guangming Xue, Xiuli Zuo, Yanqing Li, Dongsheng Li, Bo Lu, Hongliang Ren, Huicong Liu, Lining Sun

**Affiliations:** 1 School of Mechanical and Electrical Engineering, Jiangsu Provincial Key Laboratory of Advanced Robotics, Soochow University, Suzhou, China; 2 Faculty of Mechanical and Electrical Engineering, Kunming University of Science and Technology, Jingming South Road, Kunming, China; 3 Yunnan Key Laboratory of Intelligent Control and Application, Kunming, China; 4 Department of Electronic Engineering, The Chinese University of Hong Kong (CUHK), Hong Kong, China; 5 Department of Gastroenterology, Qilu Hospital, Shandong University, Jinan, Shandong China

**Keywords:** Electrical and electronic engineering, Sensors, Environmental, health and safety issues

## Abstract

In robotic-assisted surgery (RAS), traditional surgical instruments without sensing capability cannot perceive accurate operational forces during the task, and such drawbacks can be largely intensified when sophisticated tasks involving flexible and slender arms with small end-effectors, such as in gastrointestinal endoscopic surgery (GES). In this study, we propose a microelectromechanical system (MEMS) piezoresistive 3-axial tactile sensor for GES forceps, which can intuitively provide surgeons with online force feedback during robotic surgery. The MEMS fabrication process facilitates sensor chips with miniaturized dimensions. The fully encapsulated tactile sensors can be effortlessly integrated into miniature GES forceps, which feature a slender diameter of just 3.5 mm and undergo meticulous calibration procedures via the least squares method. Through experiments, the sensor’s ability to accurately measure directional forces up to 1.2 N in the Z axis was validated, demonstrating an average relative error of only 1.18% compared with the full-scale output. The results indicate that this tactile sensor can provide effective 3-axial force sensing during surgical operations, such as grasping and pulling, and in ex vivo testing with a porcine stomach. The compact size, high precision, and integrability of the sensor establish solid foundations for clinical application in the operating theater.

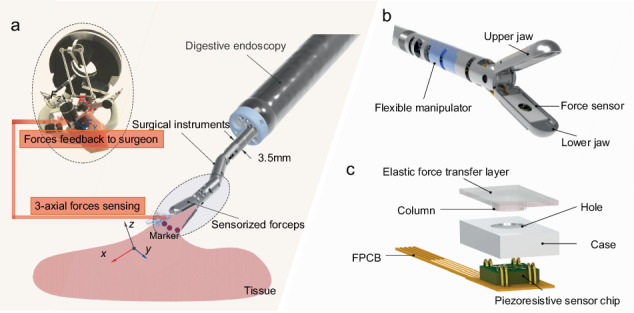

## Introduction

**Fig. 1:**
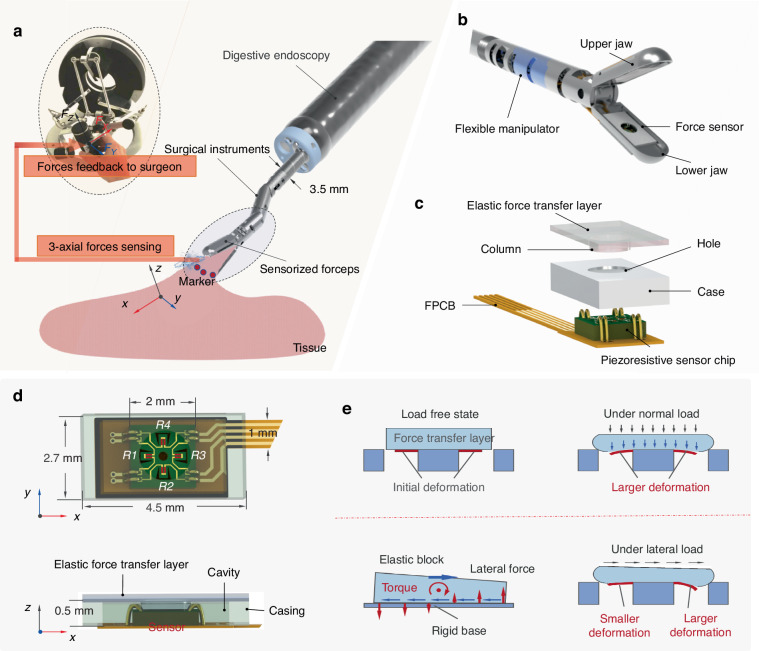
. **Schematics of the proposed 3-axial tactile sensor for GEMIS**. **a** A multi-DOF manipulator passes through endoscopy with sensorized forceps that sense 3-axial forces while manipulating. **b** The tactile sensor is integrated into the lower jaw of the GEMIS forceps. **c** An exploded view of the 3-axial tactile sensor. **d** Top view and side view of the 3-axial tactile sensor. **e** Sensing mechanism for normal force and shear force

Advancements in medical technology have positioned robotic-assisted surgery (RAS) as a crucial sector that can comprehensively enhance surgical precision and efficiency^1–6^. Recently, RAS has evolved toward single port laparoscopy^7^ (SPL) and natural orifice truncal endoscopic surgery^8,9^ (NOTES), which aim to decrease patient invasiveness, hasten recovery, and reduce the degree of complication risk^7–9^. SPL, conducted through a single, small incision, significantly reduces the number and size of surgical incisions, thereby easing patient recovery and improving cosmetic results postoperation^10^. NOTES introduces instruments via natural orifices, avoiding abdominal cuts, leading to nearly invisible scars, quicker recovery, and lower infection risks^11^. These designs include highly flexible, multiple degrees of freedom (DOFs), and miniaturized manipulators for skilled operations^1^. In NOTES, a prevalent practice involves the removal of early-stage neoplastic tissues via gastroscopy, including techniques such as endoscopic submucosal dissection (ESD). This method employs flexible endoscopes, either single- or dual-channel, to navigate the complex and winding paths of the gastrointestinal tract for surgical interventions. Figure 1a illustrates the developed surgical instrument^12,13^, which is equipped with a multi-DOF manipulator designed for gastrointestinal endoscopic procedures. This manipulator is adept at manipulating tissues through an endoscope’s 3.8 mm work channel and features an external diameter of less than 3.5 mm^12^.

However, the lack of force sensing in surgical procedures leads to a heavy reliance on visual observation and experience^4,14–17^. This gap can cause excessive force application, risking damage to sensitive tissues and increasing surgical errors; therefore, it is crucial to introduce instruments with force-sensing capabilities to improve surgical accuracy and safety^1,5^. Additionally, integrating force sensing is vital for preventing tissue slippage, thereby increasing procedural efficiency^18–20^. To address this issue, force/tactile sensors and actuators have been integrated as essential components of RAS systems. In some recent studies, sensors have been commonly integrated into the joint drive units of surgical instruments to indirectly estimate interaction forces by analyzing the responses of drivers^14,21^. Nonetheless, measurement accuracy is affected by mechanical factors such as coupling, friction, and gravity^2,22–25^; to counteract this, direct sensor placement has been explored at critical points such as the instrument’s abdominal axis, wrist joints, and tip’s direct contact areas with tissue, enhancing force measurement precision as proximity to tissue increases^2,6,23,25^. This shift highlights the growing focus on embedding tactile sensors directly onto minimally invasive surgical (MIS) instrument tips and joints for more accurate force detection^23,25^, utilizing electrical piezoresistive^2–4,6,17,26–28^, piezoelectric^29,30^, capacitive^23,25,31–34^ and optical methods^35–40^. Kim et al.^23,25^ and Lee et al.^31^ developed capacitive-based force sensors, which were integrated into surgical instruments with diameters ranging from 8 mm to 10 mm. Kuwana et al.^41^ devised a piezoresistive MEMS force sensor and incorporated it into an MIS surgical instrument with an outer diameter of 12 mm. Although these solutions address the lack of force sensing in surgical instruments, the instruments themselves remain relatively large; this highlights the ongoing need for further miniaturization of force sensing technologies. Studies conducted by Yurkewich et al.^42^, Li et al.^43^, and Suzuki et al.^44^ have employed fiber Bragg grating (FBG) technology, which measures strain by detecting shifts in the wavelength of light reflected from periodic gratings in an optical fiber. These devices were integrated at various locations on surgical instruments, such as the shaft and wrist, to enhance their ability to sense force. However, optical fibers cannot be routed within small bending radii. Additionally, the use of small and complex components in SPL and NOTES could increase manufacturing and assembly costs. These factors highlight the challenges facing fiber-based sensor solutions.

To overcome these limitations, this study introduces a miniature piezoresistive-based MEMS 3-axial tactile sensor, offering a solution for force sensing in gastrointestinal endoscopic minimally invasive surgery (GEMIS) that integrates compact sensors with high-integration and high-precision force sensing. This compact sensor, featuring a piezoresistive sensor chip equipped with four cantilever structures, is encased in a protective housing with a central aperture. An elastic layer is employed to transmit external forces, and a flexible printed circuit board (FPCB) facilitates chip bonding and signal transmission. Using MEMS fabrication techniques, a miniature piezoresistive chip was prepared. The fully encapsulated sensor can be seamlessly and extremely simply integrated into surgical forceps with an external diameter of 3.5 mm, as illustrated in Fig. 1a, b. The sensorized forceps were analyzed and calibrated for their sensing characteristics and 3-axial force dynamics. Repeatability tests demonstrated the sensor’s exceptional stability. Calibration revealed its high resolution and minimal mean relative error, highlighting significant advantages. The efficacy of the sensorized forceps was evaluated by testing their ability to grasp and pull tissue-mimetic materials and assessing the accuracy and reliability of the forces applied. An experimental demonstration on the ex vivo porcine stomach was performed to validate the effectiveness of the proposed 3-axial sensorized forceps based on the piezoresistive MEMS tactile sensor.

## Results and discussion

### Design and working principle

A prototype of the gastrointestinal endoscopic dissecting forceps^13^ incorporating the 3-axial tactile sensor is depicted in Fig. 1a. To accommodate 3 axial force manipulations, the sensorized forceps integrate a tactile sensor within the lower jaw, as illustrated in Fig. 1b. The jaw is specifically designed with a slot to securely fit the sensor. The configuration of the tactile sensor, as shown in Fig. 1c, is mainly composed of four components: an elastic force transfer layer, a stainless-steel case, an FPCB, and a piezoresistive sensor chip. The piezoresistive sensor chip, measuring 2.0 × 2.0 mm with a thickness of 0.3 mm, is designed to be sufficiently small for integration into the jaw. This sensor is bonded and wired to the FPCB, which leads the analog signal through the FPCB’s gold wire. The 2.7 × 4.5 mm case is fixed with the FPCB, which has an inner cavity that completely covers the chip. Thus, it can protect the bonding gold wires between the chip and FPCB. The elastic force transfer layer is attached to the surface of the case, whereas the column in the transfer layer is attached to the chip and in contact with the four cantilevers. Hence, external forces are allowed to make indirect contact with the sensitive cantilevers through the middle hole of the case. This rigid-flexible coupling structure enhances the sensitivity and accuracy of force signal transmission, whereas its integrated and modular architecture facilitates mass production and ensures robust environmental adaptability. In this design, an FPCB with dimensions of 1.8 m in length, 1 mm in width, and 0.1 mm in thickness has a significant advantage, as the gold flat cable can bypass the tail of the jaw and travel through the entire flexible manipulator’s inner cavity. Accordingly, it can move freely in its inner cavity when the manipulator is in operation.

The piezoresistive tactile sensor detects forces by measuring the change in resistance of piezoresistors caused by deformation under external forces. In this proposed sensor, the elastic layer functions as a cover that protects the cantilevers and interacts with the target tissue. The top view of the sensor chip is shown in Fig. 1d, with two rectangular piezoresistors positioned in the vertical direction (denoted as R2 and R4) and two more positioned in the horizontal direction (denoted as R1 and R3) (Fig. S1a, Supporting Information). Each piezoresistor consists of two rectangular piezoresistors (red blocks) with a size of 5 μm × 60 μm, and the resistances are concentrated in the stress concentration area (Fig. S1b, Supporting Information). The external forces exerted on the elastic layer are conveyed to the cantilevers via the intermediary column. In this arrangement of piezoresistive cantilevers and columns, the responses of each piezoresistive element generate distinct outputs; these allow for the independent characterization of stresses associated with external forces, specifically normal and shear forces. As depicted in Fig. 1e, under normal force, identical compressive deformations at each of the four cantilevers yield similar strain-induced changes in the resistances of the four piezoresistive elements. The uniaxial shear force applied along the X-axis leads to a strain difference in the two coaxial cantilevers, i.e., a correspondingly large increase in the resistance of R3 and a corresponding small increase in the resistance of R1, whereas the strain difference in the other two cantilevers along the Y-axes of R2 and R4 is negligible. When the forceps grasp an object, the upper and lower jaws close, and the grasped object applies a downward force on the elastic layer, resulting in compression of the tactile sensor. Subsequently, the cantilevers of the sensor deform, leading to changes in resistance. The signal wires are routed from the back of the sensor and extend through the shaft of the forceps to the end, as illustrated in Fig. 1a, b.

### Fabrication and assembly process

**Fig. 2:**
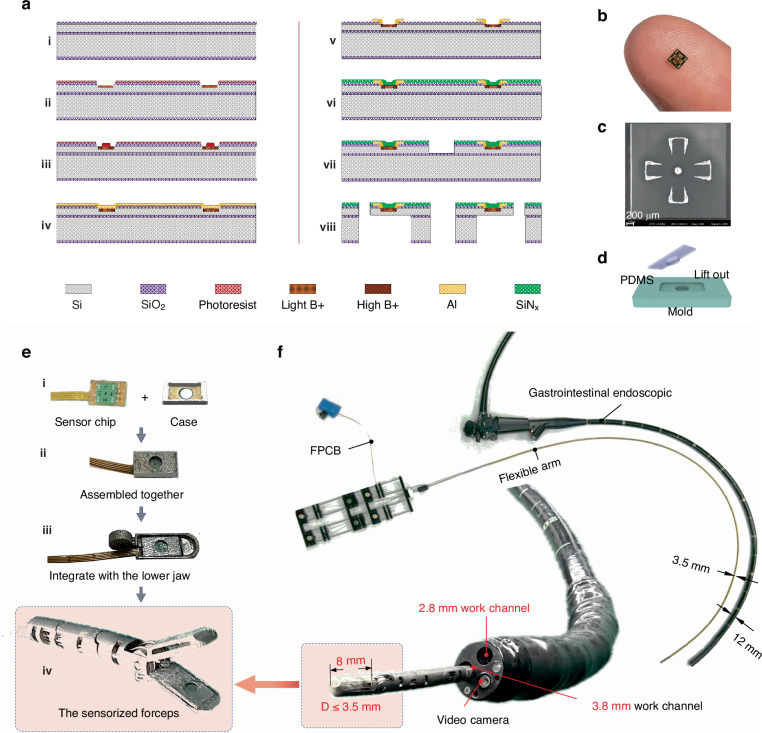
. **Fabrication of the sensor chip and assembly process of the sensorized forceps**. **a** Fabrication of the sensor chip. **b** Fabricated sensor chip on an index finger. **c** SEM image of the sensor chip. **d** Fabrication of the elastic layer. **e** i: A sensor chip fixed on the FPCB and a case; ii: Integrating the sensor chip and the case together; iii: Integrating with the lower jaw; iv: Sensorized forceps. **f** The forceps are mounted on a flexible surgical arm with a diameter of 3.5 mm, passing through the 3.8 mm working channel of the gastrointestinal endoscope

Figure 2a illustrates the sensor chip detailed fabrication process. The procedure commences with an n-type, (100)-oriented silicon-on-insulator (SOI) wafer, which is utilized as the initial substrate and features a device layer thickness of 5 µm. Initially, SiO_2_ layers were thermally grown on both sides of the wafer. The subsequent steps included a photolithography process on the wafer’s front side to delineate the piezoresistors. This was followed by the implantation of boron ions at a 5 × 10^14^ cm^−2^ dosage. Rapid thermal annealing (RTA) was then applied to activate the dopants, thereby forming piezoresistors. A subsequent boron ion implantation was carried out at a 2 × 10^15^ cm^−2^ dosage, followed by an RTA step to establish ohmic contacts. Aluminum was then sputtered and patterned to a thickness of 700 nm, facilitating the metallization required for interconnections with the piezoresistors. Precise regulation of the diffusion temperature and duration enabled consistent and efficient doping, achieving less than 2% variability in the mean value across four cantilevers within a single batch (Fig. S1c, Supporting Information). Plasma-enhanced chemical vapor deposition (PECVD) was employed to deposit a 1 µm layer of silicon nitride (SiN_x_), which served to offset the compressive stress within the SiO_2_ layer and protect the surface electrodes. The fabrication continued with the opening of contact pads and the patterning and etching of cantilevers. Backside deep reactive ion etching (DRIE) was conducted to release the cantilever structure down to the buried oxide (BOX) layer. The buried SiO_2_ layer was subsequently removed via reactive ion etching. The final sensor chip, measuring 2.0 × 2.0 × 0.3 mm^3^, is depicted in Fig. 2b and is positioned on an index finger. The cantilever structures are clearly visible in the scanning electron microscope (SEM) image shown in Fig. 2c.

In addition to the piezoresistive sensor chip, the elastic force transfer layer is also crucial, as it transmits external excitation to sensitive cantilevers and serves as an encapsulation. A stainless-steel mold, as shown in Fig. 2d and Fig. S2 (Supporting Information), is needed to form the column structure; thus, it can pass through the case’s hole (Fig. 1c) and contact the sensor’s cantilevers. The height of the column is crucial to ensure that the column and the cantilevers maintain proper contact when load-free. The height of the column is determined by the following factors: the height of the sensor chip (300 μm) and the height of the case (500 μm). Hence, the height of the column was determined to be 200 μm. Polydimethylsiloxane (PDMS) with a mixture ratio of 10:1 (polydimethylsiloxane polymer base:polymerization agent) was poured into the machined mold. The mixture was placed in a vacuum box for 10 min to eliminate bubbles and then heated for 15 min at 100 °C in an oven. After that, the samples were lifted to obtain the elastic layer, resulting in a Young’s modulus of approximately 2.61 MPa for the transfer layer^45^ (Table S1, Supporting Information). Finally, a final elastic layer with dimensions of 4.5 × 2.7 mm and a central column of 1.6 × 0.2 mm was produced (Fig. S2, Supporting Information).

Adhering to the outlined specifications, the sensor-integrated jaw was meticulously crafted. The main body and housing are constructed from 304 stainless steel and are endowed with a Young’s modulus of 200 GPa, ensuring robustness and durability. The gripping section of the jaw body spans a length of 8.0 mm, and upon closure, the total external diameter of the conjoined jaws remains below the 3.5 mm threshold (Fig. S3, Supporting Information). This compact design facilitates seamless insertion through the endoscope’s 3.8 mm diameter working channel. Coupling this sensor-equipped jaw with an external 3.5 mm flexible manipulator enables precise navigation and operation under endoscopic control.

The piezoresistive sensor chip is meticulously bonded to the FPCB at a height of 0.1 mm. A stainless-steel sheet, which is also 0.1 mm thick, reinforces the sensor’s stationary region to prevent deformation and maintain sensor integrity (Fig. S4, Supporting Information). Figure 2e (i) shows the sensor fixed on the FPCB and a case. The case is placed over the sensor chip while ensuring that the sensor’s sensitive unit is fully visible in the case’s hole, as shown in Fig. 2e (ii). The encasing measures 2.7 × 4.5 × 0.6 mm^3^, tailored to the slot into the lower jaw with precision. Once inserted, the tactile sensor is anchored in place via silicone gel, ensuring a secure and stable assembly, as shown in Fig. 2e (iii). Finally, the upper and lower jaws are connected to the flexible manipulator via a hinge pin, as shown in Fig. 2e (iv). The assembled forceps ensure that only the flexible elastic layer and the main body of the jaw encounter biological tissues. Both PDMS and stainless steel, which are used in their construction, exhibit excellent biocompatibility. This methodical assembly ensures that the sensorized forceps are equipped with a 3-axial force-sensing capability and are primed for complex surgical procedures.

Figure 2f shows a gastrointestinal endoscopic device featuring two work channels: a 2.7 mm channel for an electric knife (dual knife) and a 3.8 mm channel for a flexible manipulator, along with a light source and lens. During procedures, the dual knife and flexible manipulator are inserted through these channels to the target area to perform electrodissection (electrocoagulation) and grasping tasks. The light source illuminates the confined surgical field, and the lens provides visual feedback. The sensorized forceps, integrated into the flexible manipulator tip, facilitate 5-DOF movements, including grasping, pitching, yawing, rolling, and advancing^12,13^. Table 1 compares this sensor’s integrated instruments with those used in previous studies, demonstrating that the surgical digestive endoscopy forceps integrated with this sensor have the smallest external diameter, measuring only 3.5 mm.

**Table 1:** Comparison of different force perception methods in MIS

Study	Method and location	Instrument	D (mm)
Kim et al.^23,25^	Capacitive-based transducers integrated into the grasper	S-Surge Surgical Robot	8
Lee et al.^31^	Capacitive-based sensor integrated into wrist and instrument base	RAVEN-II	10
Kuwana et al.^41^	Piezoresistive-based sensor integrated into grasper	MIS laparoscopic grasper	12
Yurkewich et al.^42^	FBGs-based sensor integrated into distal shaft and gripper	MIS arthroscopic grasper	4.57
Li et al.^43^	FBGs-based sensor integrated into articulated wrist	Palpation probe	4
Suzuki et al.^44^	FBGs-based sensor near the tip of forceps	Bilateral micro-operation system	4
This work	Piezoresistive-based sensor integrated into the forceps	GEMIS	3.5

### Force-sensing characterization and calibration

**Fig. 3:**
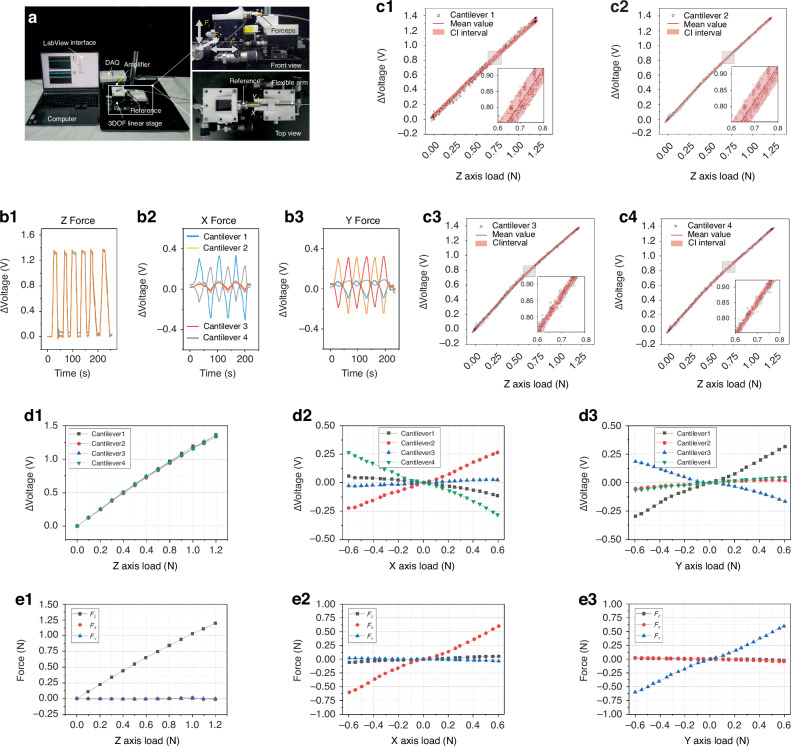
. **Characterization and calibration of the sensorized forceps**. **a** Entire view of the experimental setup for the calibration of the developed forceps. **b** Repeated curves of the cantilevers as a function of the normal force and lateral force. **c** Each cantilever’s voltage versus external force along the Z axis results, with a 95% confidence interval evaluated over 6 attempts. **d** Voltage data measured by the 3-axial tactile sensor at the developed forceps with respect to the external 3-axial forces measured by the reference sensor. **e** Calibrated 3-axial force of the jaw with respect to the reference 3-axial forces

After successfully fabricating the sensor-integrated forceps, a calibration experimental setup was established, as depicted in Fig. 3a. A commercial 6-axis force/torque sensor (Nano 17, ATI) mounted on a 3-DOF linear stage served as the reference for the measurements. The reference sensor was connected to an amplifier and a data acquisition system (NI-USB 6210, DAQ). Analog signals from the sensor were conditioned by electrical circuitry, where they were filtered and amplified before digitization by the DAQ and subsequent transmission to the computer. The sensor electrical circuit employs a wheatstone bridge with three fixed resistors, each matched to the piezoresistor’s initial resistance (Fig. S5, Supporting Information). External forces alter the piezoresistor’s resistance, unbalancing the bridge. This imbalance induces a voltage change, which is then amplified by AD620. An AD705 serves as a buffer to the amplifier, stabilizing the output voltage at 2 V when the bridge is balanced. The circuit’s final output is directed to the DAQ (NI-USB 6210) and recorded through the data acquisition procedure (Fig. S6, Supporting Information). This setup allows for the capture of voltage signals from the four piezoresistors, enabling the computation of resistance changes and corresponding voltage fluctuations in each cantilever. In the front and top views of the calibration setup, as shown in Fig. 3a, the 3-DOF stage exerts three orthogonal forces, *F*_*X*_, *F*_*Y*_, and *F*_*Z*_, onto the lower jaw of the forceps via its triaxial motion capabilities through one 3D printing jig. During the experiments, the measured voltage data and the reference data were recorded simultaneously.

To evaluate the performance of the tactile sensor, normal force and shear force tests, including loading and unloading, were conducted. During the process, the experiments were repeated approximately 90 times along the Z axis. Figure S7 (Supporting Information) shows the raw data of the four cantilevers without a filter, which confirmed the outstanding repeatability of the sensor. In Fig. S8 (Supporting Information), a fast response time of less than 5 ms is observed under an impact force from a temporal test. Applying the Kalman filter method (which is more suitable than the Butterworth low-pass filter method in this context) to the raw voltage data results in a smoother curve (Fig. S9, Supporting Information). Figure 3b1 shows six repeated curves of the cantilevers as a function of the normal force from 0 to 1.2 N, whereas Fig. 3b2, b3 display three repeated curves of the four cantilevers as a function of the lateral force along the X and Y axes from −0.6 to 0.6 N, respectively. To best meet the design requirements of surgical instruments, efforts have been made to achieve a grasping force of 0.77 N and a traction force of 0.71 N^12^. While there is potential for further increasing the calibrated normal force, a normal force of 1.2 N is sufficient to prevent the lower jaw from slipping when the shear force is increased to 0.6 N, thus ensuring effective calibration. Although the 0.6 N shear force is slightly below the target value, the data still meet the requirements for procedures such as endoscopic submucosal dissection. The relationships between the voltages and the normal force of the four cantilevers are presented in Fig. 3c1, c2, c3, c4 with 95% confidence intervals. The series of repeated tests confirmed the sensor’s outstanding repeatability, as evidenced by the majority of voltage readings aligning within the prescribed confidence intervals. This consistency provides credible data underpinning the sensor’s calibration process.

To measure the quantitative values of forces, bridging the gap between the voltage variances of four cantilevers and the 3-axial forces on the basis of proper calibration is essential. Figure 3d1, d2, d3 shows the tendencies of the four cantilevers’ voltage data of the forceps based on the reference data. In the graphs, the data points for the voltages correspond to the mean of repeated measurements, ranging from 0 to 1.2 N along the Z-axis and from −0.6 to 0.6 N along the X- and Y-axes. As shown in Fig. 3d1, the four voltages are changed in the same direction. The voltages increase with increasing external force from 0 to 1.2 N. At the same time, the voltage data for the four cantilevers exhibit almost uniform variations. Externally applied forces in the X-direction induce significant voltage changes in cantilevers 2 (V_2_) and 4 (V_4_), which are aligned with the X-axis, whereas cantilevers 1 (V_1_) and 3 (V_3_), oriented along the Y-axis, exhibit minimal voltage variations, as shown in Fig. 3d2. The trends in voltage shifts for cantilevers 2 and 4 are entirely antithetical. The modest voltage fluctuations observed in cantilevers 1 and 3 are attributed primarily to assembly inaccuracies. Conversely, the shear forces applied in the Y direction yield an inverse response, as shown in Figure 3d3. As a result, the tendencies of the measured voltages with respect to the external 3-axial forces are consistent with the explanation provided by the working principle.

For the purpose of calibrating the 3 axial forces from the voltage data after filtering, establishing a calibration matrix was imperative. Given the observed good linearity of the voltage responses of the four cantilevers under the application of triaxial forces, this matrix was deduced via the linear least squares method (see Text S1, Supporting Information), which provides a correlation between the external 3-axial forces and the corresponding voltage readings. The calibration matrix $A$ is calculated as:

1 $$A=\left[\begin{array}{cc}\begin{array}{cc}-0.1411 & 0.48225\\ 0.25225 & 1.01904\\ 0.33861 & 1.28641\end{array} & \begin{array}{cc}-0.2460 & 0.77293\\ 0.10931 & -1.3922\\ -2.4574 & 0.88168\end{array}\end{array}\right]$$

Thus, the 3-axial forces $${\left[\begin{array}{ccc}{F}_{X} & {F}_{Y} & {F}_{Z}\end{array}\right]}^{T}$$ can be calculated through the equation $$A\cdot {\left[\begin{array}{cc}\begin{array}{cc}{V}_{1} & {V}_{2}\end{array} & \begin{array}{cc}{V}_{3} & {V}_{4}\end{array}\end{array}\right]}^{T}$$. Consequently, the calibrated 3-axis forces measured by the sensorized forceps with respect to the reference force are illustrated in Fig. 3e, in which e1 represents the Z-axis force, e2 represents the X-axis force, and e3 represents the Y-axis force. The experimental findings enabled the determination of the force resolution for the tactile sensor, which was established as 0.66 mN, 0.61 mN, and 0.28 mN for the respective axes. Figure S10 shows the relative error ratio of the maximum force of the forceps in the calibration results. The mean relative error was calculated to be 1.15%, 2.43%, and 1.18% of the full-scale output (FSO) force range, corresponding to each axis. The sensor performance metrics are listed in Table 2. For a detailed calculation description, see texts S1, S2, and S3 (Supporting Information).

**Table 2:** Performance of the developed 3-axial tactile sensor with sensorized forceps

Quantity	Value			Unit
	*X*-axis	*Y*-axis	*Z*-axis	
Force range	0.6	0.6	1.2	N
Force resolution	0.66	0.61	0.28	mN
Mean relative error	1.15	2.43	1.18	% of FSO

*FSO* Full scale output force range

### Experimental validations of sensorized forceps

In RAS, the use of forceps can be categorized into two main scenarios: grasping and pulling. Grasping primarily involves the application of normal forces, whereas pulling primarily involves the application of shear forces. Two distinct types of materials are used to simulate two radically different tissues within the gastrointestinal tract. PDMS with a doping ratio of 30:1 (Young’s modulus of 0.86 MPa, Table S1, Supporting Information) was used to simulate tumor tissues, such as gastric adenocarcinoma or gastrointestinal stromal tumors. These tissues are generally harder and have a different texture than the gastric mucosa. To simulate the conditions of the gastric mucosa during gastrointestinal surgery, thermoplastic rubber (TPR) was selected as the demonstration model for system verification.

**Fig. 4:**
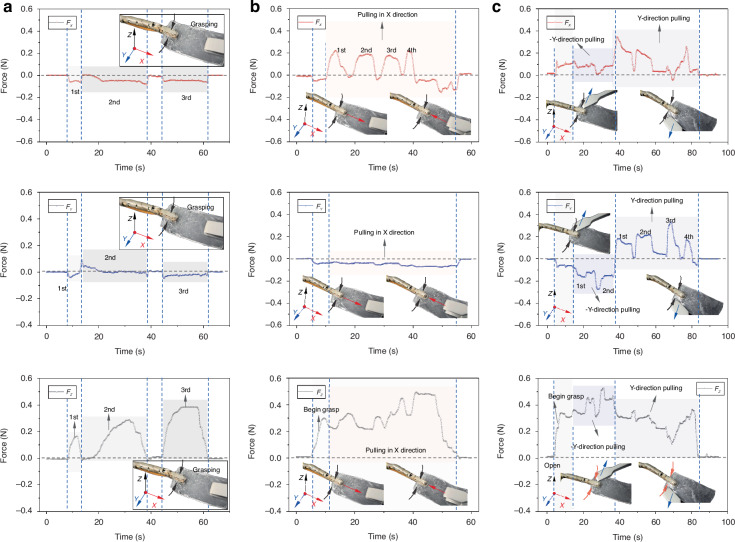
. **Responses of the sensorized forceps while they grasp simulated tumor tissue (PDMS)**. **a** Grasping three times. **b** Grasping and pulling processes along the X direction. **c** Grasping and pulling processes along the Y direction

Initially, the forceps perform three grasping actions on the simulated tumor tissue, followed by a pulling action along the X-axis. The forceps subsequently grasp the simulated tumor tissue and move it along the Y-axis, thereby evaluating the ability to detect forces in different directions. During this process, data collection occurs at a sampling frequency of 1000 Hz, and the obtained three-axis force values are presented in Fig. 4. Importantly, as the forceps incrementally engage with the simulated tumor tissue, the concurrent generation of both shear and normal forces ensues, as shown in Fig. 4a. In three repeated grasping trials, the values of ${F}_{Z}$ are 0.17 N, 0.28 N, and 0.39 N, respectively, whereas the absolute values of ${F}_{X}$ and ${F}_{Y}$ are each less than 0.1 N. The forceps also exhibit commendable responsiveness to pulling actions on tissues in the X and Y directions. During the X direction pulling tests, the variations in ${F}_{X}$ are clearly distinguishable in four repeated trials, with values of 0.22 N, 0.2 N, 0.19 N, and 0.23 N, respectively. Throughout the entire pulling process, the forceps continuously grasp the tissue, resulting in ${F}_{Z}$ values consistently above 0.2 N, with a maximum value of 0.48 N. The ${F}_{Y}$ values remain relatively stable, ranging between −0.03 N and −0.08 N. However, during the pulling of tissue in the Y direction, traction on the tissue does not occur purely along the Y-axis but is accompanied by a component in the X direction, resulting in a significant response in ${F}_{X}$ with each action, as shown in Fig. 4c.

**Fig. 5:**
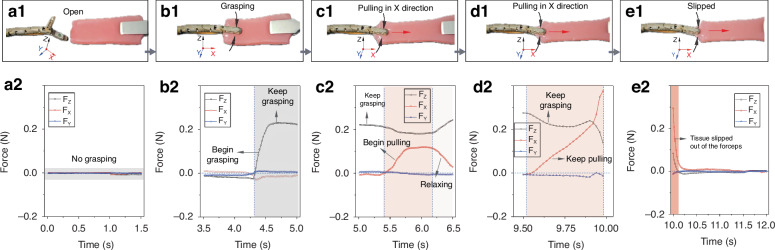
. **Responses of the sensorized forceps while they grasp the simulated gastric mucosa (TPR)**. **a** No grasping. **b** Grasping. **c** Grasping and pulling processes along the X direction. **d** Grasping and keeping pulling. **e** Keep pulling, and the tissue slips out of the forceps

Owing to the softer nature of simulated gastric mucosa (TPR), it is easier to discern the applied actions from deformation during grasping and pulling. The grasping and pulling actions are illustrated in Fig. 5a1–e1, with the force value responses at various stages shown in Fig. 5a2–e2. As the forceps grasp the simulated gastric mucosa, the three-axis forces begin to be displayed in Figure 5b2, with ${F}_{Z}$ showing the most significant increase from 0 N to 0.22 N. ${F}_{X}$ and ${F}_{Y}$ exhibit slight variations, and their absolute values remain predominantly below 0.03 N. Upon pulling in the X direction, the ${F}_{X}$ value increases to 0.11 N; when the pulling stops, the ${F}_{X}$ value decreases. As pulling continues, ${F}_{X}$ increases further to 0.28 N, which is even greater than the grasping force of 0.22 N. During the pulling process, the tissue begins to slip within the forceps, and the amount of simulated mucosa gripped by the forceps continuously decreases, as shown in Fig. 5d2. When a sudden decrease in ${F}_{X}$ occurs, as shown in Fig. 5e2, the simulated mucosa has lost its effective grip within the forceps and needs to be regripped.

**Fig. 6:**
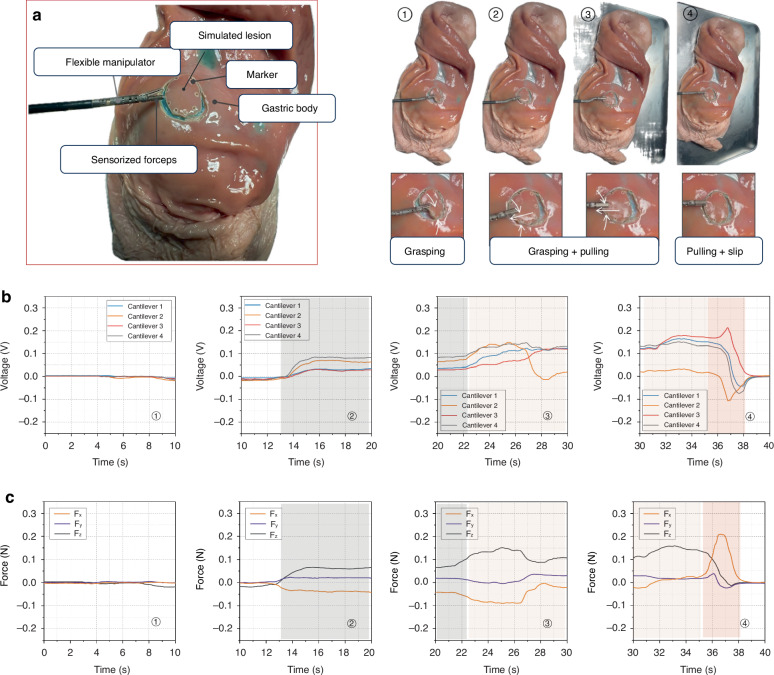
. **Experimental validation of the sensorized forceps while grasping circumcised gastric mucosa**. **a** Overview of the developed forceps and the circumcised gastric mucosa within the gastric body, alongside the complete experimental process. **b** Voltage response of the four cantilevers recorded throughout the experiment. **c** Calibrated force response from the forceps captured during the experiment

Although TPR is sufficiently soft to simulate the gastric mucosa, it still differs significantly from the actual gastric mucosa. Consequently, we prepared a fresh porcine stomach and randomly selected an area on the gastric body for marking and circumcising. We then used the sensorized forceps to grasp and pull the circumcised gastric mucosa, assessing the effectiveness of these forceps in a real porcine stomach test. The ex vivo experiments on a porcine stomach are depicted in Fig. 6a. During the experiment, an assistant positioned the flexible arm at an appropriate location on the stomach body, while the operator controlled the arm’s movement and the forceps’ opening and closing with their main hand to grasp and pull the precircumscribed lesion. The grasping and pulling actions are illustrated from ① to ④, with the voltage values and force values responding at various stages shown in Fig. 6b, c. Throughout the grasping and pulling process, changes in three-dimensional forces occur, particularly at the beginning of grasping, the beginning of pulling, and when slippage occurs. The maximum grasping force during the process is approximately 0.17 N, which is significantly lower than the forces observed when grasping the PDMS and TPR. Unlike the TPR, the value of ${F}_{X}$ inversely increases when the actual gastric mucosa is grasped, and the absolute value of *F*_*X*_ decreases as pulling continues. This is primarily due to the irregular shape of the gastric mucosa. Compared with ${F}_{X}$ and ${F}_{Z}$, the force ${F}_{Y}$ is smaller. Feedback on the entire process of force changes is provided to the operator, allowing them to feel the progression of these changes and determine the actions of grasping and pulling.

## Conclusion

This study introduces a compact piezoresistive MEMS 3-axial tactile sensor for GEMIS. This tactile sensor, characterized by its innovative design, incorporates a piezoresistive chip with four cantilever structures housed within a protective casing featuring a central aperture. An elastic force transfer layer is utilized for the transmission of external forces, whereas an FPCB ensures efficient chip bonding and signal transmission. The meticulous application of MEMS process flow techniques facilitated the creation of a miniature piezoresistive chip, measuring only 2.0 × 2.0 × 0.3 mm^3^. The fully encapsulated sensor, with overall dimensions of 4.5 × 2.7 × 0.6 mm^3^, demonstrates the feasibility of its integration into surgical forceps with an external diameter of 3.5 mm, offering a straightforward and seamless incorporation process.

The study further delves into a comprehensive analysis and calibration of the sensorized forceps, focusing on their sensing characteristics and 3-axial force dynamics. Through repeated tests, the tactile sensor shows exceptional stability, with calibration results revealing its superior resolution as high as 0.28 mN in ${F}_{Z}$ and minimal average relative error down to 1.18% of FSO. These findings underscore the sensor’s significant advantages, particularly in terms of its performance and reliability.

Moreover, the practical application and effectiveness of the sensorized forceps were assessed through tests involving the manipulation of tissue-mimetic materials and ex vivo manipulation of porcine gastric tissue. These tests, aimed at evaluating the accuracy and reliability of both grasping and pulling forces, demonstrated the sensor’s potential in enhancing the precision and efficacy of surgical procedures. In future work, we will further focus on optimizing and minimizing the sensor design to increase its versatility and broaden its potential applications in NOTES, especially in DREAMS (dual-arm robotic endoscopic assistants for minimally invasive surgery). Machine learning algorithms or models, particularly deep neural networks, are used to calibrate and decouple the sensor, thereby systematically enhancing its decoupling accuracy. Additionally, animal clinical trials will be conducted to assess the feasibility and effectiveness of the sensor in a clinical environment.

## Experimental section

### Description of the amplifier circuit for analog signals

Figure S5 illustrates the amplifier circuit employed for the sensor, with a detailed schematic of the individual circuit shown on the right. The circuit operates with a 5 V power supply, incorporates three fixed resistors within the Wheatstone bridge, and is calibrated to match the resistance of the initial piezoresistor value. When subjected to external forces, the resistance of the piezoresistor changes, thereby disturbing the balance of the Wheatstone bridge. The resultant voltage output from the bridge is subsequently amplified by the AD620 operational amplifier, which has a gain factor of 19.93 and an RG resistance of 2611 Ω. To achieve an initial output voltage of 2 V in the balanced state, an AD705 is employed as a follower to the amplifier chip. The output of the circuit is then directed to the analog input of a data acquisition system (DAQ), specifically the NI-USB 6210. Consequently, voltage signals from the four piezoresistors are available, enabling the computation of resistance changes (ΔR) and voltage variations in each direction.

### Description of the signal acquisition system based on LabView

To ensure real-time and accurate voltage data acquisition from the sensorized forceps and the standard sensor, a real-time data acquisition and display interface was developed via LabVIEW, as shown in Fig. S6a. This interface allows real-time display of voltage changes in the force-sensing forceps due to the piezoresistive effect. The LabVIEW display interface juxtaposes the signal data from the sensorized forceps and the ATI Nano17, facilitating a more intuitive comparison of their signal responses. This not only enhances the display quality but also enables the synchronous acquisition of time-domain signals from both sensors. The detailed acquisition interface is shown in Fig. S6b, where the upper section displays the force/torque information from the standard sensor, and the lower section shows the unfiltered voltage signals from the sensorized forceps.

### Preparation of gastric corpus-mimic lesions

First, the inner and outer walls of the pig stomach were rinsed with clean water, and the stomach was placed into a stainless-steel tray. The bottom of the tray is equipped with a negative electrode plate required for the high-frequency electric knife. The coagulation function of the electric knife was used to mark the area around the gastric body before circumcision. After marking, an electric knife was used to cut along the outer edge of the marks, and the mucosal tissue below the inner wall of the circumscribed area was cut to simulate the lesion.

## Supplementary information

Supporting information

Grasping and Pulling the Simulated Tumor Tissue in X- and Y- Direction

Grasping and Pulling the Mimic Gastric Mucosa in X-Direction

## Data Availability

All data needed to evaluate the conclusions in the paper are presented in the paper and/or the Supplementary Materials. Additional data related to this paper may be requested from the authors.
