# Supplementary material for: A piezoresistive-based 3-axial MEMS tactile sensor and integrated surgical forceps for gastrointestinal endoscopic minimally invasive surgery
Source: Microsyst Nanoeng. 2024 Sep 27;10:141. doi: 10.1038/s41378-024-00774-6 (PMC11427553; doi:10.1038/s41378-024-00774-6)
Supplement: Supplementary file 1 — Supporting information [file 41378_2024_774_MOESM1_ESM.docx]

Supporting information for

**A Piezoresistive-based 3-axial MEMS Tactile Sensor and Its Integrated Surgical Forceps for Gastrointestinal Endoscopic Minimally Invasive Surgery**

*Cheng Hou ^a, b, c^, Huxin Gao ^d^, Xiaoxiao Yang ^e^, Guangming Xue ^a^, Xiuli Zuo ^e^, Yanqing Li ^e^,*

*Dongsheng Li ^a^, Bo Lu ^a,^ *, Hongliang Ren ^d^, Huicong Liu ^a,^ *, and Lining Sun ^a,^ **

*^a^ School of Mechanical and Electrical Engineering, Jiangsu Provincial Key Laboratory of Advanced Robotics, Soochow University, Suzhou, China*

*^b^ Faculty of Mechanical and Electrical Engineering, Kunming University of Science and Technology, Jingming South Road, Kunming, 650500, China*

*^c^ Yunnan Key Laboratory of Intelligent Control and Application, Kunming, 650500, China*

*^d^ The Department of Electronic Engineering and Shun Hing Institute of Advanced Engineering, The Chinese University of Hong Kong (CUHK), Hong Kong*

*^e^ The Department of Gastroenterology, Qilu Hospital, Cheeloo College of Medicine, Shandong University, Jinan, Shandong, 250012, China*

* Corresponding authors. E-mail: blu@suda.edu.cn; [hcliu078@suda.edu.cn](mailto:hcliu078@suda.edu.cn); lnsun@hit.edu.cn

**This file includes:**

Text S1 to S3

Table S1

Figures S1 to S10

Other Supplementary Material for this manuscript includes the following:

Movies S1 and S2


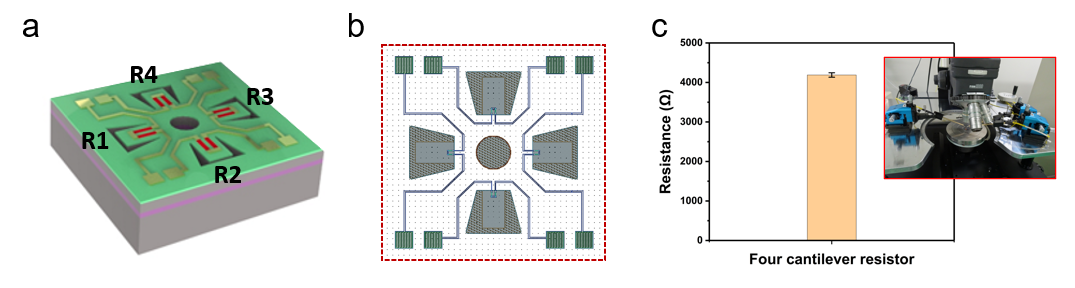


Figure S1. Schematic of the sensor chip and piezoresistive resistances characterization. (a) Schematic of the sensor chip and the locations of R1-R4. (b) The layout of the sensor chip. (c) Four cantilevers’ resistance in a single batch.


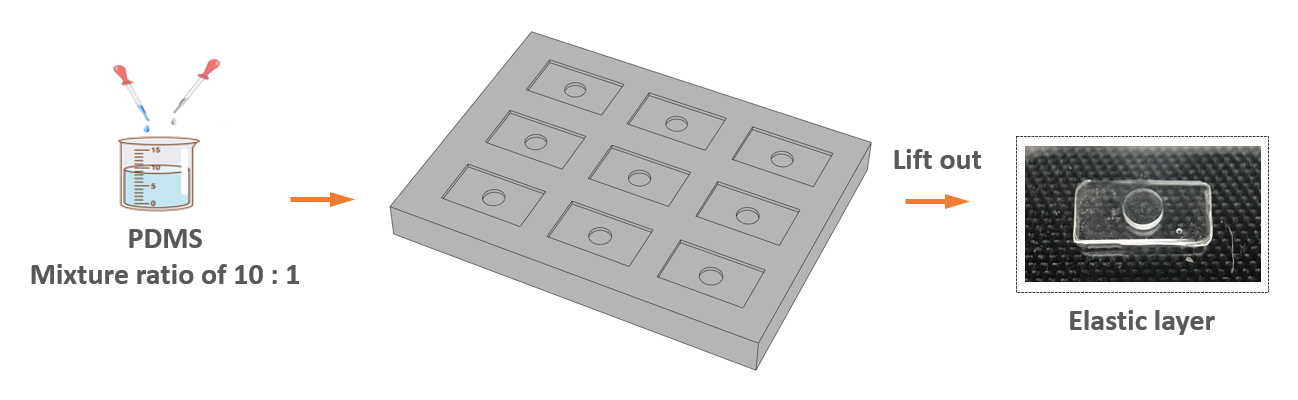


Figure S2. Schematic depiction of the fabrication process for the elastic force transfer layer.


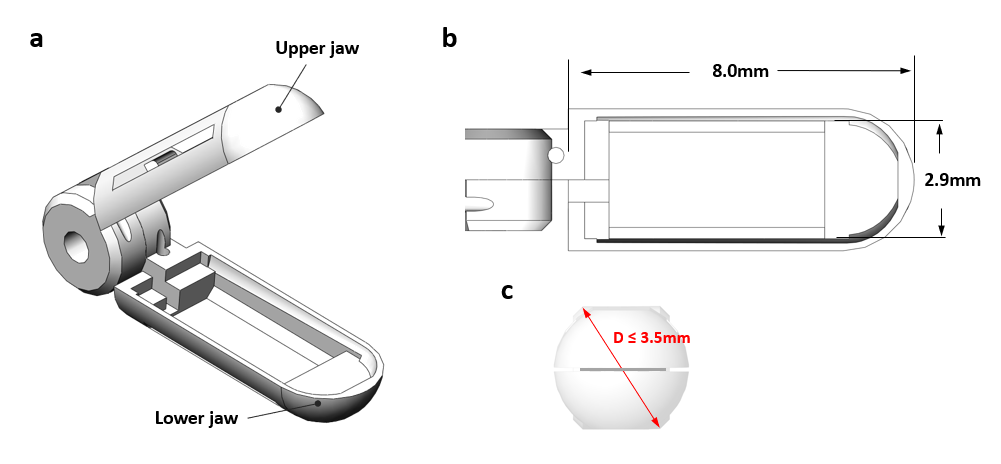


Figure S3. Schematic representation of the forceps. (a) Displays the forceps without any sensor attachments. (b) Details the dimensions of the slot designed for sensor integration. (c) The external diameter of the forceps when closed is less than 3.5 mm.


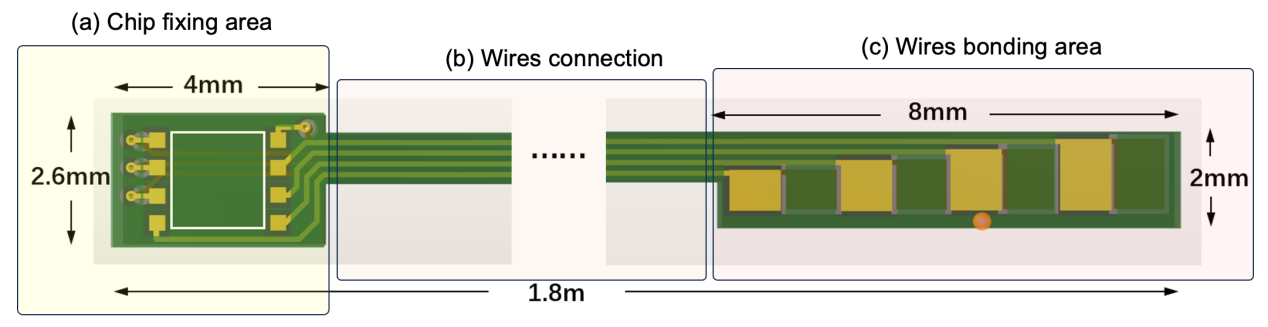


Figure S4 Schematic diagram of the FPCB. (a) Sensor chip fixing area, mounted in the jaw’s slot. (b) Wires connection area, passing through the inner cavity of the flexible arm. (c) Wires bonding area, connected to the circuit board through bonding wires.


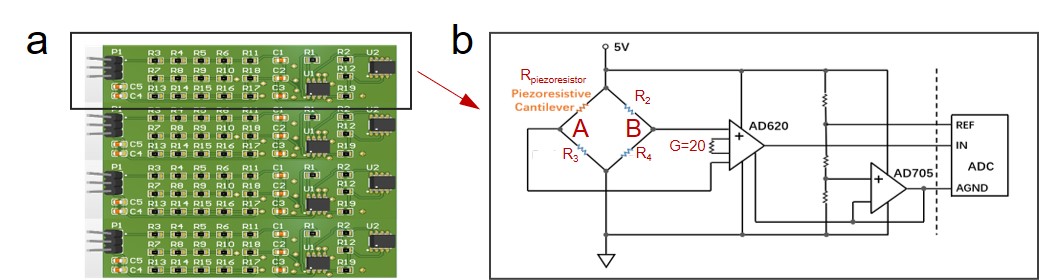


Figure S5. Schematic of the electrical circuit. (a) Complete circuit board diagram for the sensor chip. (b) Schematic circuit diagram for each piezoresistive resistance.


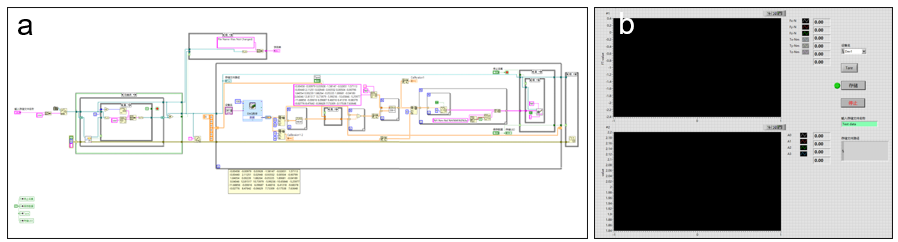


Figure S6. (a) Signal Acquisition Block Diagram. (b) Signal acquisition interface based LabVIEW includes the reference sensor’s data (upper) and four cantilevers’ voltage (lower).


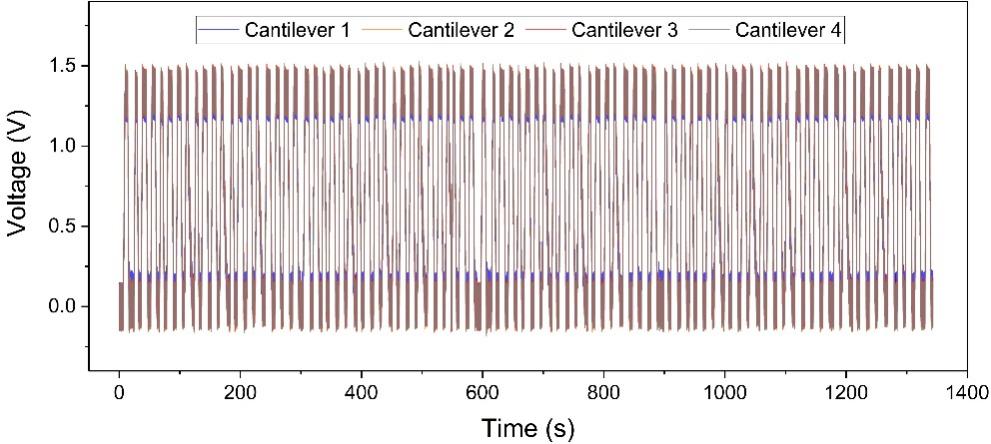


Figure S7. The voltage output of the four cantilevers in the sensor during repeated tests under normal force, with a maximum normal force of 1.2 N.


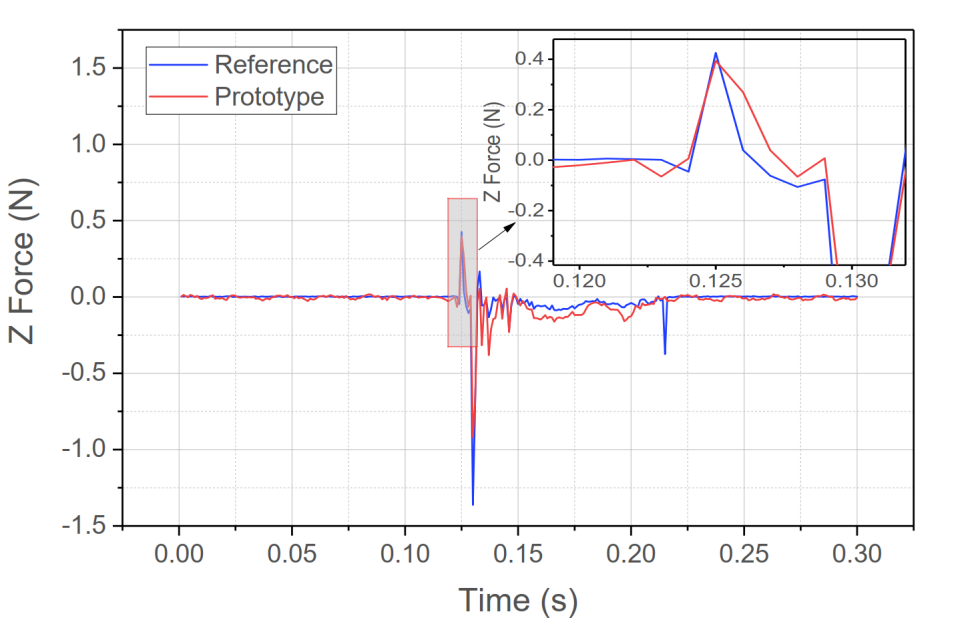


Figure S8. Dynamic response of this fabricated sensor measured in Z-axial (blue line: reference sensor, red line: this fabricated sensor).


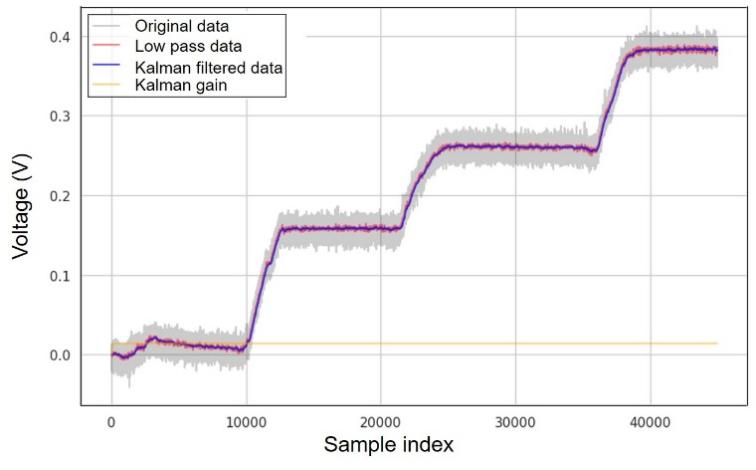


Figure S9. The raw voltage data subjected to low-pass filtering, Kalman filtering and the resulted Kalman gains, respectively.


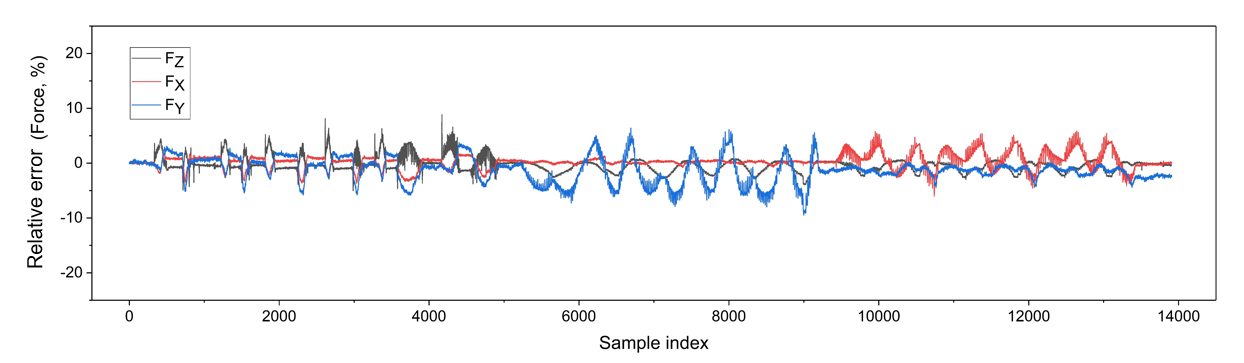


Figure S10**.** The relative errors ratio of maximum force of the sensorized forceps in calibration results.

**Text S1. Calibration of the linear least squares method**

Due to the relatively good linear relationship between the sensor's input and output, the output voltage of each channel can be obtained through linear superposition. The relationship between the output voltage vector $\left[ \begin{matrix} \begin{matrix} V_{1} & V_{2} \end{matrix} & \begin{matrix} V_{3} & V_{4} \end{matrix} \end{matrix} \right]^{T}$ and the input force vector $\left[ \begin{matrix} F_{X} & F_{Y} & F_{Z} \end{matrix} \right]^{T}$ is given by:

$\left[ \begin{matrix} \begin{matrix} V_{1} \\ V_{2} \end{matrix} \\ \begin{matrix} V_{3} \\ V_{4} \end{matrix} \end{matrix} \right]= \left[ \begin{matrix} \begin{matrix} \begin{matrix} C_{11} & C_{12} & C_{13} \end{matrix} \\ \begin{matrix} C_{21} & C_{22} & C_{23} \end{matrix} \end{matrix} \\ \begin{matrix} \begin{matrix} C_{31} & C_{32} & C_{33} \end{matrix} \\ \begin{matrix} C_{41} & C_{42} & C_{44} \end{matrix} \end{matrix} \end{matrix} \right]\cdot\left[ \begin{matrix} F_{X} \\ F_{Y} \\ F_{Z} \end{matrix} \right]$ (1)

where C is a 4 × 3 calibration matrix with constant elements, $\left[ \begin{matrix} \begin{matrix} V_{1} & V_{2} \end{matrix} & \begin{matrix} V_{3} & V_{4} \end{matrix} \end{matrix} \right]^{T}$ is the output voltage vector in volts (V), and $\left[ \begin{matrix} F_{X} & F_{Y} & F_{Z} \end{matrix} \right]^{T}$ is the input three-dimensional force vector in newtons (N). The least squares solution to the matrix equation yields the calibration matrix C:

$\boldsymbol{C=}{\boldsymbol{F}\boldsymbol{U}^{\boldsymbol{T}}\boldsymbol{(U}\boldsymbol{U}^{\boldsymbol{T}}\boldsymbol{)}}^{\boldsymbol{-1}}$ (2)

Based on the standard sensor output obtained from static calibration experiments and the voltage output of the forceps, we can determine C. Next, we compute the generalized inverse matrix of C, and by using $\boldsymbol{A=}{\boldsymbol{(}\boldsymbol{C}^{\boldsymbol{T}}\boldsymbol{C)}}^{\boldsymbol{-1}}\boldsymbol{C}^{\boldsymbol{T}}$, we can derive the coefficient matrix A. Consequently, the input force vector of the sensorized forceps can be obtained as $\boldsymbol{F=A}\boldsymbol{\cdot}\boldsymbol{U}$.

**Text S2. Calculation of the force resolution**

The output of the force sensor is converted into a voltage through the Wheatstone bridge, which is then digitized by the NI 6210. Several key aspects to calculate the resolution should be considered:

First, relationship between the output and force. Secondly, NI 6210 input range and resolution. The NI 6210 has a 16-bit ADC, which means it can digitize signals into 2^16^=65,536 distinct levels. Assuming the input voltage range is ±10 V, the voltage resolution is 20/65,536 V. At last, converting Voltage Resolution to Force Resolution. By combining the voltage resolution with the relationship between the output voltage and force, we can calculate the force resolution.

$Force resolution=Voltage resolution\times{Maximum force}/{Maximum output voltage}$ (3)

In this scenario, the force resolution can be calculated. Taking the Z-axis direction as an example, when subjected to an external force of 1.2N, the maximum voltage output is 1.3V. Therefore, its resolution can be calculated to be 0.28mN.

**Text S3. Calculation of the mean relative error**

The average relative error was calculated to assess the accuracy of the sensor after calibration. The relative error for each test point was determined based on the full-scale output of the sensor. The steps for this calculation are as follows:

For each test point i, the average relative error over N test points is computed as

$Average {Relative error}_{i}= \frac{1}{N}\sum_{i=1}^{N} \frac{{|F}_{actual,i}-F_{meatured,i}|}{F_{max}}$ (4)

where $F_{actual,i}$ is the actual force and $F_{meatured,i}$ is the measured force. This metric provides a quantitative measure of the sensor's accuracy post-calibration, highlighting the discrepancy between the actual and measured forces relative to the full-scale output.

**Table S1 Young’s modulus E and Poisson’s ratio ν of the PDMS with different mixture ratio**

|  | PDMS  (5:1) | PDMS  (10:1) | PDMS  (20:1) | PDMS  (30:1) | PDMS  (40:1) | PDMS  (50:1) | PDMS  (60:1) |
| --- | --- | --- | --- | --- | --- | --- | --- |
| E | 2.8MPa | 2.61MPa | 1.16MPa | 0.86MPa | 0.71MPa | 0.62MPa | 0.57MPa |
| *ν* | 0.33 | 0.33 | 0.33 | 0.33 | 0.33 | 0.33 | 0.33 |
